# PPARα-mediated peroxisome induction compensates PPARγ-deficiency in bronchiolar club cells

**DOI:** 10.1371/journal.pone.0203466

**Published:** 2018-09-13

**Authors:** Srikanth Karnati, Gani Oruqaj, Harshavardhan Janga, Srinu Tumpara, Claudia Colasante, Paul P. Van Veldhoven, Nancy Braverman, Adrian Pilatz, Thomas J. Mariani, Eveline Baumgart-Vogt

**Affiliations:** 1 Institute for Anatomy and Cell Biology II, Division of Medical Cell Biology, Justus Liebig University, Giessen, Germany; 2 Laboratory of Lipid Biochemistry and Protein Interactions, KU Leuven, Leuven, Belgium; 3 Depts. of Human Genetics and Pediatrics, McGill University-Montreal Children’s Hospital Research Institute, Montreal, Canada; 4 Department of Urology, Pediatric Urology and Andrology, Justus Liebig University Giessen, Giessen, Germany; 5 Division of Neonatology and Pediatric Molecular and Personalized Medicine Program, University of Rochester Medical Center, Rochester, New York, United States of America; Tokyo University of Agriculture, JAPAN

## Abstract

Despite the important functions of PPARγ in various cell types of the lung, PPARγ-deficiency in club cells induces only mild emphysema. Peroxisomes are distributed in a similar way as PPARγ in the lung and are mainly enriched in club and AECII cells. To date, the effects of PPARγ-deficiency on the overall peroxisomal compartment and its metabolic alterations in pulmonary club cells are unknown. Therefore, we characterized wild-type and club cell-specific PPARγ knockout-mice lungs and used C22 cells to investigate the peroxisomal compartment and its metabolic roles in the distal airway epithelium by means of 1) double-immunofluorescence labelling for peroxisomal proteins, 2) laser-assisted microdissection of the bronchiolar epithelium and subsequent qRT-PCR, 3) siRNA-transfection of PPARγand PPRE dual-luciferase reporter activity in C22 cells, 4) PPARg inhibition by GW9662, 5) GC-MS based lipid analysis. Our results reveal elevated levels of fatty acids, increased expression of PPARα and PPRE activity, a strong overall upregulation of the peroxisomal compartment and its associated gene expression (biogenesis, α-oxidation, β-oxidation, and plasmalogens) in PPARγ-deficient club cells. Interestingly, catalase was significantly increased and mistargeted into the cytoplasm, suggestive for oxidative stress by the PPARγ-deficiency in club cells. Taken together, PPARα-mediated metabolic induction and proliferation of peroxisomes via a PPRE-dependent mechanism could compensate PPARγ-deficiency in club cells.

## Introduction

Peroxisomal proliferator-activated receptors (PPARs) are ligand-activated transcription factors belonging to the nuclear hormone receptor family [1]. The first member of this nuclear receptor family was identified and cloned during the search for a receptor that could mediate the proliferation of peroxisomes in hepatocytes in the liver of rats treated with hypolipidemic drugs (e.g. clofibrate) [2]. So far, three different PPAR isoforms have been identified: PPARα, PPARβ/δ, PPARγ [1, 2]. All PPAR isotypes display distinct tissue-specific expression and modulate cell-type specific gene-transcription in response to external factors. Most of the literature regarding the role of PPARs in the lung has focused on the functional characterization of PPARγ, because it was shown to play a central role in anti-inflammatory processes in this tissue. PPARγis expressed in different pulmonary cell types and its functions were shown to be cell-type specific [3]. The highest expression of PPARγ was found in club and AECII cells where it was suggested to regulate epithelial cell differentiation and to control airway inflammatory processes [3]. All PPARs exhibit a similar molecular mode of action and a partially overlapping lipid ligand spectrum with different binding sensitivities. After binding their preferred ligand, they form heterodimers with the nuclear receptor RXR, are targeted to the nucleus, and subsequently bind to DNA-response elements in the target genes known as peroxisome proliferator response elements (PPREs). PPREs are present in many genes regulating the transcription of proteins involved in the biogenesis and metabolism of peroxisomes.

Peroxisomes are involved in a variety of metabolic pathways, including the synthesis of membrane lipid precursors, e.g. cholesterol and plasmalogens, and polyunsaturated fatty acids as well as the β-oxidation of fatty acids, the degradation of various toxic and proinflammatory mediators and the metabolism of reactive oxygen species (ROS) [4]. During these processes a number of lipid-intermediates are generated, which could act as intracellular ligands for PPARs and we have previously suggested a peroxisome/PPAR loops in the control of lipid homeostasis in the heart [5]. The high number of peroxisomes in club and AECII cells containing high levels of peroxisomal β-oxidation enzymes indicates that these organelles could indeed play a role in the homeostasis of lipid ligands for PPARs also in the lung. In this respect, it is of interest that PPARγand peroxisomes display the same distribution pattern in the lung [4].

To dissect the functional role of PPARγin the lung epithelium, Mariani’s group generated a club cells-specific PPARγknockout mice using the cre-loxP system under the control of CC10 promoter [6, 7]. Club cell-specific PPARγknockout-mice (ccsPPARγKO) exhibited insufficient postnatal lung maturation and epithelial cell differentiation. Moreover, PPARγ-deficient bronchial epithelial cells showed a drastic increase of the expression of the *Pex7* gene, that encodes a cytoplasmic receptor targeting proteins with N-terminal peroxisomal targeting sequence 2 (PTS2) to the peroxisomal matrix [6]. However, so far no information is available on the effects of PPARγ-deficiency on the regulation of the peroxisomal compartment in airway epithelial cells. Therefore, in this study, we have used lung-tissue derived from ccsPPARγKO mice to investigate the overall effects on the expression of genes coding for peroxisomal proteins in distal airways. Our results reveal strong peroxisome proliferation and induction of all major peroxisomal pathways, such as increased biogenesis, β-oxidation and ether lipid synthesis in PPARγ-deficient club cells. Additionally, triglycerides accumulated and distinct fatty acids were elevated. Further, the mRNAs for PPARα and its mitochondrial target genes were increased, suggesting the compensation of the PPARγ-deficiency in club cells by the upregulation of PPARα-dependent signaling. The modulation of the peroxisomal metabolism in PPARγ-deficient club cells might be necessary to protect the airway epithelium against oxidative and lipotoxic stress and to prevent chronic inflammation in distal airways.

## Materials & methods

### Materials

DNase I, oligo (dT) 12–18 primers, superscript II reverse transcriptase, TOTO-3-iodide were purchased from Invitrogen (Karlsruhe, Germany), Tween 20, Hoechst 33342, GW9662, were from Sigma-Aldrich (Deisenhofen, Germany). The Dual-Luciferase Reporter Assay System (Cat. E1910) was bought from Promega (Mannheim, Germany). The RNeasy Plus Kit and the PPAR Reporter Kit (Cat. CCS-3026L) was obtained from Qiagen (Hilden, Germany). Maxima SYBR Green qPCR Master Mix (Cat. K0243) was purchased from Thermo Scientific (Dreieich, Germany). Primers for quantitative reverse transcriptase (RT)-PCR were synthesized by Eurofins (Ebersberg, Germany); Mouse genes and proteins were named according to the official NIH nomenclature throughout the manuscript.

### Animals and tissue material

Lung tissue sections were prepared from nine animals that were 8–9 week old as previously described [6]: WT (PPARγfloxed/floxed, CC10-Cre-), conditional knockout mice (KO) (PPARγfloxed/floxed, CC10-Cre+) and CC10-Cre (WT, CC10-Cre+)."The methods of animal experiments were carried out in strict accordance with the recommendations in the Guide for the Care and Use of Laboratory Animals of the Harvard Medical School (HMS). All experimental protocols were approved by veterinary and laboratory licensing committee of the Harvard Medical School". Thomas J Mariani generated these mice at HMS [6]. Adult mice were euthanized by CO2 narcosis, followed by exsanguination. Neonatal mice were anesthetized with CO2 and euthanized by decapitation.

### Immunofluorescence (IF) and quantification

The detailed procedure for lung perfusion and paraffin embedding of the animals was described previously by Simon et al [6]. Paraffin sections (2–3 μm) were cut with a Leica RM2135 rotation microtome and processed for double immunofluorescence as described [4, 8–10]. Dilutions of the primary and secondary antibodies used are listed in Table 1. Fluorescent images were taken from sections stained with peroxisomal antibodies (green) and marker proteins (CC10 or α-tubulin) analyzed using a Leica TCS SP5 confocal laser scanning microscope (Leica GmbH, Wetzlar, Germany). Images were captured with a 63x objective, setting at Airy 1, 1x zoom and 10 times sampling. All images were processed with Adobe Photoshop CS5 and quantified using ImageJ software (National Institutes of Health).

**Table 1:** List of antibodies used in this study.

***Primary antibodies***			
**Cell type-specific antigens**	**Species ab raised in (AB)**	**Dilution (IF)**	**Supplier**
Club cell marker protein 10 (CC10), mouse	Goat, polyclonal	1:50	Santa Cruz Biotechnology Inc., Heidelberg, Germany, Cat. no: sc-25555
α-tubulin, porcine	Mouse, monoclonal	1:2,000	Santa Cruz Biotechnology Inc., Heidelberg, Germany, Cat. no: sc-8035
ABC transporter D3 (ABCD3/PMP70), rat	Rabbit, polyclonal	1:1,000	Gift from Alfred Völkl, Dept. of Anatomy and Cell Biology, Heidelberg, Germany; see reference: (Beier et al. 1988)
Peroxin 14 (Pex14p), mouse	Rabbit, polyclonal	1:2,000	Gift from Denis I. Crane, School of Biomol. Biophys. Sci., Griffith Univ., Nathan, Brisbane, Australia; see reference: (Maxwell et al. 2003)
Catalase (CAT), mouse	Rabbit, polyclonal	1:4,000	Gift from Denis I. Crane (address see above); see reference: (Maxwell et al. 2003)
Acyl-CoA oxidase 1 (ACOX1), rat	Rabbit, polyclonal	1:1,000	Van Veldhoven et al. 1994
Thiolase, rat	Rabbit, polyclonal	1:1,000	Antonenkov et al. 1999
***Secondary antibodies***			
**Secondary detection system used**	**Host**	**Dilution**	**Supplier**
anti-Rabbit-IgG AlexaFluor488	Donkey	1:1,000	Molecular Probes/Thermofischer, Cat. no: A21206
anti-Goat-IgG AlexaFluor594	Chicken	1:1,000	Molecular Probes/Thermofischer, Cat. no: A11058
**Counterstaining of nuclei for IF**			
Hoechst 33342 (1 μg/ml) nucleic acid staining	-	-	Molecular Probes/Thermofischer, Cat. no: 33342
TOTO-3 nucleic acid staining, 1:1,000	-	-	Molecular Probes/Thermofischer, Cat. no: T-3604

### Laser capture microdissection (LCM)

WT and KO lung tissues were embedded directly into a cryo-preservative solution (Optimal Cutting Temperature, OCT, Tissu-tek) in freezing molds and placed in liquid nitrogen. 7–10 μm thick cryosections were mounted on 1mm polyethylene naphthalate membrane-covered slides (PALM Microlaser Technologies GmbH, Bernried, Germany), and processed as previously described [9, 11]. The bronchiolar epithelial cells were identified by their anatomic location and morphology, marked on computer screen, and dissected by LCM (*PALM*Microlaser Technologies).

### RT-PCR of total RNA of bronchiolar epithelial cells isolated by LCM

Total RNA was isolated from microdissected bronchial epithelial cells using the RNeasy Plus Qiagen kit. The reverse-transcription was performed as previously described [12].

### Quantitative reverse transcriptase polymerase chain reaction (qRT-PCR)

Specific primers were designed with the Primer3 online software (http://bioinfo.ut.ee/primer3-0.4.0/) and ordered online from Eurofins MWG Operon (http://www.operon.com). Primers used in this study are listed in Table 2. Quantitative RT-PCR analysis was carried out by using the SYBR premix (Thermo Scientific Dreieich, Germany) on an iCycler PCR machine (Bio-Rad, Heidelberg, Germany), according to the manufacturer´s instructions. Normalization for cDNA quantity was done using *Hprt* control primer for each template. The fold change and the normalized values for different mRNAs of *Pparγ* WT and *Pparγ* KO were calculated by using the ddCT method. All RT-PCR experiments were performed three times using the total RNA from three distinct isolation experiments. Graphs were made using the GraphPad prism software version 5 and the statistical significance was determined using the unpaired t-test.

**Table 2:** List of primers used in this study.

Gene target	Gene bank accession no.	Sence primer (5’-3’)	Antisence primer (5’-3’)	PCR product (bp)	Ann. Temp. °C
***Abcd3***	NM_008991.2	TCAGAATGGGACGCTCATTGA	TGGCAGCGATGAAGTTGAATAA	86	57.0
***Acaa1***	AK143187.1	CAATGAACTGAAGCGTCGTG	CACCACTGTGGCACTCTCTG	141	59.0
***Acadvl***	AK076037.1	TGGATCAATTTGCCACCGTG	TCAGAGAAGGCACATGACCT	152	58.0
***Acox1***	NM_015729.2	CCGCCACCTTCAATCCAGAG	CAAGTTCTCGATTTCTCGACGG	86	61.0
***Acox2***	NM_001161667.1	ACGGTCCTGAACGCATTTATG	TTGGCCCCATTTAGCAATCTG	125	57.9
***Agps***	AK_031049	TGTCCTCCGTGTCTGTTCCT	CATGGTACAACCTGCCCTTC	141	59.4
***Cat***	BC013447	GGAGAGGAAACGCCTGTGTGA	GTCAGGGTGGACGTCAGTGAAA	103	64.0
***Cpt1***	AK149688.1	TCCAAGTATCTGGCAGTCGA	AGTCCATTTTCCTTCCGTGC	128	57.0
***Cact***	AK152436.1	GTGGCTTTGCAGGGATCTTC	GGTGACTCCTTCTTCTCGGA	143	58.0
***Cc10***	AK145844.1	GCCTCCAACCTCTACCATGA	GGACTTGAAGAAATCCTGGGC	107	59.5
***Gnpat***	AK_010896.1	TGAGGACGTGCAAGCCTTTG	TCCAGAAGCTGACGGGTGAA	121	58.0
***Mfp1***	NM_023737.3	AATACAGCGATACCAGAAGCCA	CCAGCTCTAGTCCTCCTCCA	71	59.6
***Mfp2***	NM_008292.4	TTAGGAGGGGACTTCAAGGGA	TCGCCTGCTTCAACTGAATCG	119	59.8
***Pex13***	NM_023651.4	TGGATATGGAGCCTACGG	CGGTTAAAGCCCAAACCATT	81	57.9
***Pex14***	NM_019781.2	GCCACCACATCAACCAACTG	GTCTCCGATTCAAAAGAAGTCCT	97	59.0
***Pex5***	NM_008995.2	AATGCAACTCTTGTATCCCGA	GGCGAAAGTTTGACTGTTCAATC	91	58.5
***Pex7***	NM_008822.2	GAACACTGTGTGTTGGGTGTG	CTGCTTTTCAGGTTCGGAAG	138	59.5
***Phyh***	AK146753.1	TGCCAGTTTCCAGCCTGAAC	CCTGGGAGCACAACCAGACA	125	57.0
***Pparα***	AK081709.1	TCCTTTCTGAATGGGCACTT	TTAACATTGGGCCGGTTAAG	125	55.0
***Pparβ***	AK089913.1	GCGGGCTCTAGAATTCCATC	CCGTCTTCTTTAGCCACTGC	137	59.4
***Pparγ***	BC021798.1	TTTTCAAGGGTGCCAGTTTC	CATGGACACCATACTTGAGCA	128	56.0
***α-tubulin***	AK164335.1	CCTTCTTCAGTGAGACAGGAGC	TCCTTGCCTGTGATGAGCTG	139	57.9

### C22 cell culture and *Pparγ* siRNA transfection of C22 cells

The C22 cells were maintained as previously described [12]. Briefly, the cells were maintained in permissive conditions (Dulbecco’s modified Eagle’s medium (DMEM) maintained in 2% fetal bovine serum (FBS), 100 U/ml penicillin, 1% streptomycin, 250 μg/ml amphotericin B, 5 μg/ml transferrin, 100 U/ml γ-INF, 10 μg/ml insulin, 0.025 μg/ml epidermal growth factor, 7.5 μg/ml endothelial cell growth supplement, 40 nmol/ml endothelin-1, 0.36 μg/ml hydrocortisone, 20 ng/ml T3) at 33°C. For siRNA experiments, C22 cells were cultured at 37°C and siRNA for *Pparγ knockdown* was incubated with InCella ScreenFect, a siRNA Transfection Reagent (InCella, Germany) in a 12-well culture plate and allowed to form a complex for 15 min at room temperature. The complex was added to the cell suspension for each well (final siRNA concentration of 10 nM). Seventy two hours after transfection, cells were collected by centrifugation (200 g for 5 min at RT) and the pellet processed for further RNA isolation and qRT-PCR analysis. The supernatants were collected and subjected to H_2_O_2_ assay as per manufacturer instructions.

### PPAR-γ antagonist (GW9662) treatment

C22 cells were seeded for 24 h in 12-well plates, and treated with 5 μM GW9662 (Sigma-Aldrich Chemie GmbH), for 24 h. After treatment, cells were processed for RNA isolation and subsequently for qRT-PCR analysis. The supernatant was used for H_2_O_2_ assay.

### Transfection and dual-luciferase reporter gene assay

The PPRE luciferase reporter assay experiments were performed by using the Dual-Luciferase Reporter Assay System together with a PPAR Signal Reporter Kit from Qiagen according to the protocol and the manufacturer´s instructions. Briefly, C22 cells were cultured overnight in a 12-well plate. Cells were either transfected with *Pparγ* siRNA or treated with GW9662 reagents for certain time-points. After 24 hours from the siRNA transfection or before treatment with GW9662 cells were transfected with the PPRE vector or the negative control vector using 4 μl Trans IT1LT-1 transfection Reagent (Cat. MIR2300) purchased from Mirus (VWR, Darmstadt, Germany) according to the manufacturer´s instructions. Then, cells were lysed with luciferase lysis reagent (Promega), and firefly or Renilla luciferase activity was measured 24 h after transfection with the luminometer (Lumat LB 9507 from BERTHOLD Technologies, Pforzheim, Germany).

### Measurements of hydrogen peroxide (H_2_O_2_) in vitro

C22 cells were transfected with *Pparγ* siRNA or treated with GW9662 for certain time points and collected by centrifuging at 250 g for 5 min. After 72 hours from siRNA transfection or 24 hours after GW9662 treatment cells were processed for H_2_O_2_ analysis. In vitro H_2_O_2_ concentrations were measured using the Amplitefluorimetric hydrogen peroxide assay kit (ABD Bioquest, Sunnyvale, CA) according to the manufacturer’s instructions. Briefly, a 50 μl cell supernatant was incubated with an equal volume mixture of Amplite Red peroxidase substrate and horseradish peroxidase for 30 min at room temperature. The signal at 690 nm in a 96-well microtiter plate was detected and recorded by a microplate reader.

### Lipid analysis

Total lipid extracts [13] from lung tissue (20–25 mg) were analyzed for phospholipids (ashing followed by ammonium molybdate/malachite green assay) [14], triglycerides [15] and plasmalogens [16]. An aliquot of the lipid extract, corresponding to ~50 nmol phospholipid, was fortified with tricosanoic acid as internal standard and subjected to acid hydrolysis (BF3/methanol), followed by GC-MS analysis of the methylesters (Trace GC-MS, Thermo Finnigan; equipped with an automated cold-on-column injector and high oven temperature device connected to a Zebron 50 30 m column (Phenomenex); EI+ mode at 35 eV). Total ion current signals were related to the internal standard signal and converted to nmol fatty acid using experimentally obtained relative response factors [17].

### Statistical analysis

All values are expressed as means ± SEM where n = 3 or 4. An unpaired Student’s t-test wasused to assess the difference between two groups. Image J was used for quantification of staining. Differences were consideredstatistically significant when *p≤0.05; **p≤0.01; ***p≤0.001.

## Results

### Peroxisomal biogenesis and ROS metabolizing enzyme are increased in PPARγ-deficient club cells

**Fig 1:**
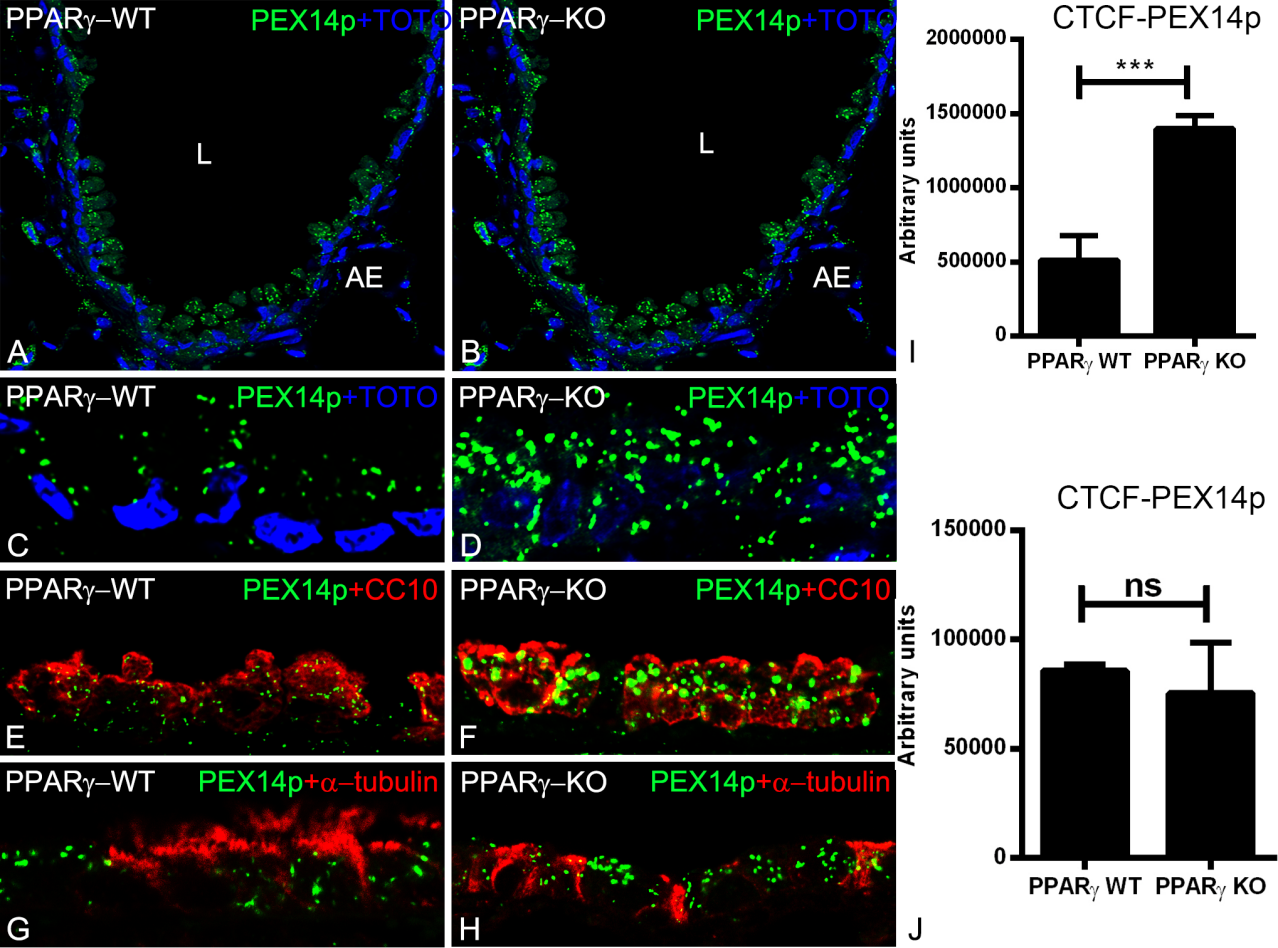
Immunofluorescence detection of the peroxisomal protein PEX14p, involved in organelle biogenesis (A-H) in paraffin-embedded lung tissue sections of wild-type (1A, 1C, 1E, 1G) and PPARγ-deficient club cells (1B, 1D, 1F, 1H). Representative lower (1A and 1B) and higher magnifications (1C and 1D) stained for PEX14p showing the cross sections of terminal bronchioles of the mouse lung. Double-IF for club cell specific marker protein CC10 (1E) and ciliated cell-specific marker protein α-tubulin (1G) together with PEX14p in WT in comparison to PPARγ-deficient club cells (1F, 1H). Note that, PPARγ-deficiency increased the overall number of peroxisomes and PEX14p protein abundance (1I). Further, staining for PEX14p showed higher amounts in club cells in comparison to ciliated cells in ccsPPARγKO cells, but neighboring ciliated cells still contain PEX14p with similar amounts (1J). Corrected total cell fluorescence (CTCF) quantification of staining for PEX14p (1I and 1J). Values ± SEM represent the mean of CTCF quantified from images obtained from 3 independent experiments using Image J software. **P ≤0.01; ***P ≤0.001; ns, not significant. Counterstaining of nuclei was performed with TOTO-3 iodide in 1A-1D. 1L: lumen of the bronchiole; AE: alveolar epithelium. Bars represent 1A-1B: 100 μm; 1C-1H: 25 μm.

**Fig 2:**
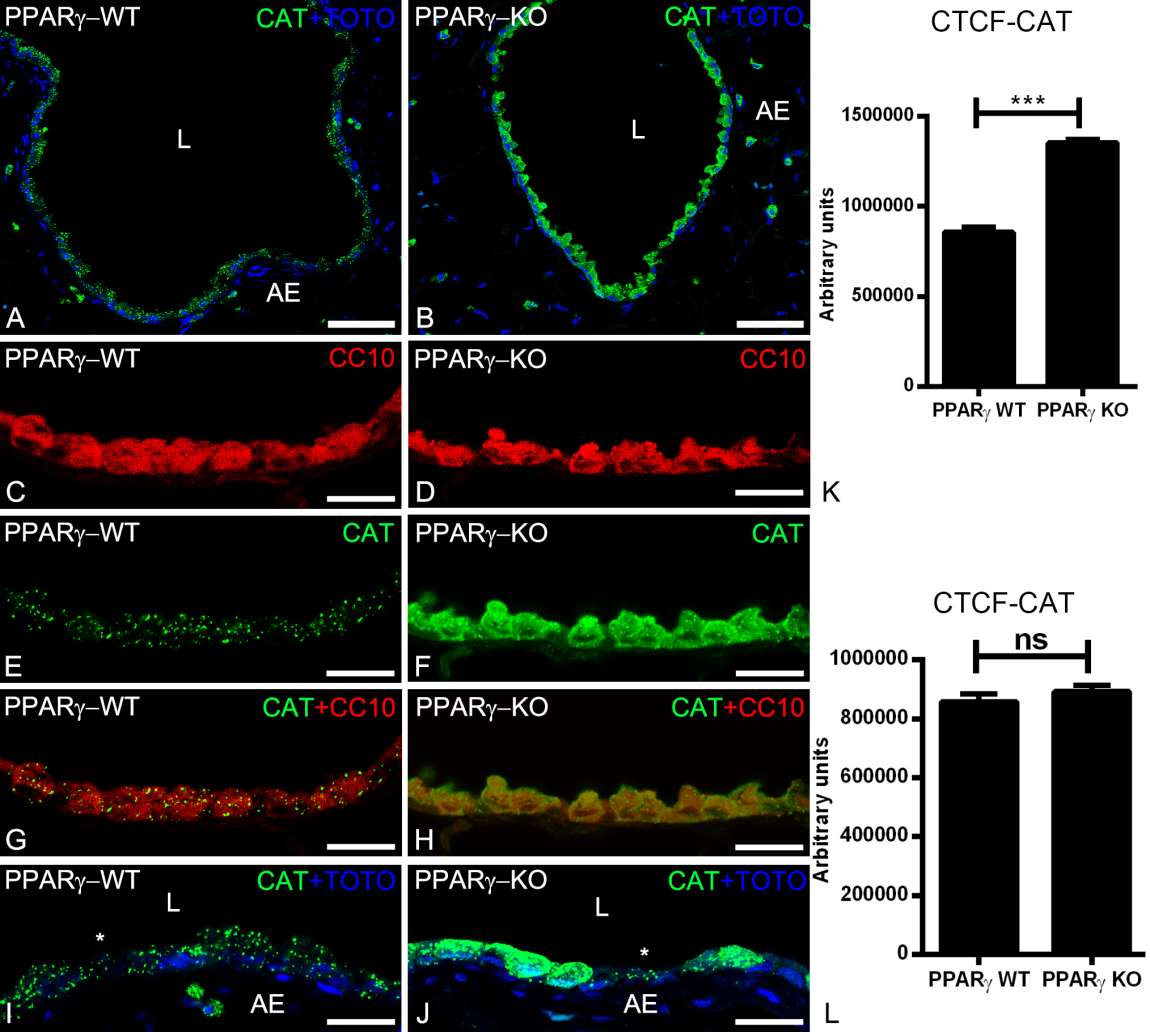
Immunofluorescence detection of the peroxisomal marker enzyme catalase in paraffin-embedded lung tissue sections of wild-type (2A, 2C, 2E, 2G, 2I) and PPARγ-deficient club cells (2B, 2D, 2F, 2H, 2J). Note that, PPARγ-deficiency increased the catalase protein abundance. Moreover, the catalase is partially mistargeted into the cytoplasm (2F, 2H, 2J). Club cells-specific protein CC10 stained club cells (2C, 2D) and higher magnifications (2E and 2F) stained for catalase showing the cross sections of terminal bronchioles of the mouse lung. Cytoplasmic staining of catalase was observed in CC10 positive club cells (2G, 2H). Staining for catalase showed punctuate staining pattern in higher amounts in club cells in comparison to ciliated cells (*) in WT animals, whereas it is present in cytoplasm (in addition to peroxisomes) in ccsPPARγKO club cells, but neighboring ciliated cells still contain punctuate catalase with similar amounts (2I, 2J, 2K, 2l). Corrected total cell fluorescence (CTCF) quantification of staining for CAT in club cells (2K) and ciliated cells (2I). Values ± SEM represent the mean of CTCF quantified from images obtained from 3 independent experiments using Image J software. **P ≤0.01; ***P ≤0.001; ns, not significant. Counterstaining of nuclei was performed with TOTO-3 iodide in 2A, 2B, 2I, 2J. 2L: lumen of the bronchiole; AE: alveolar epithelium. *: ciliated cell; Bars represent 2A-2B: 100 μm; 2C-2H: 25 μm.

The best marker protein to visualize the abundance of peroxisomes at the light microscopic level is PEX14p. PEX14p is a component of the docking complex of the peroxisomal membrane that is required for the translocation of matrix proteins into the peroxisomes [4]. As shown by IF analysis, the PEX14p antibody immunodecorated peroxisomes in the bronchiolar epithelium of wild type animals (Fig 1A, 1C, 1E and 1G). The PEX14p labelling intensity and the number of peroxisomes were significantly increased in the PPARγ-deficient club cells suggesting a proliferation of the peroxisomal compartment with active peroxisomal biogenesis (Fig 1B, 1D, 1F, 1H and 1I). In addition to PEX14p, also the peroxisomal antioxidative enzyme catalase, which is highly abundant in club cells of the bronchiolar epithelium, was significantly increased by PPARγ-deficiency (Fig 2A, 2B, 2E, 2F and 2K). Moreover, catalase in PPARγ-deficient club cells was partially mistargeted to the cytoplasm (Fig 2B, 2F, 2H and 2J). Double-IF with epithelial cell marker proteins (CC10 for club cells or α-tubulin for ciliated cells) showed that, PPARγ-deficiency in club cells induced the upregulation of PEX14p (Fig 1E–1H) and CAT (Fig 2G–2J) solely in club cells but not in ciliated cells (Figs 1J and 2L)) suggesting a PPARγ-mediated cell-specific reaction. Appropriate WT-Cre control sections obtained by separate breeding did not show any differences in PEX14p intensity or peroxisome abundance in comparison to PPARγKO animals (S2 Fig).

### The peroxisomal β-oxidation machinery is increased by the PPARγ-deficiency

**Fig 3:**
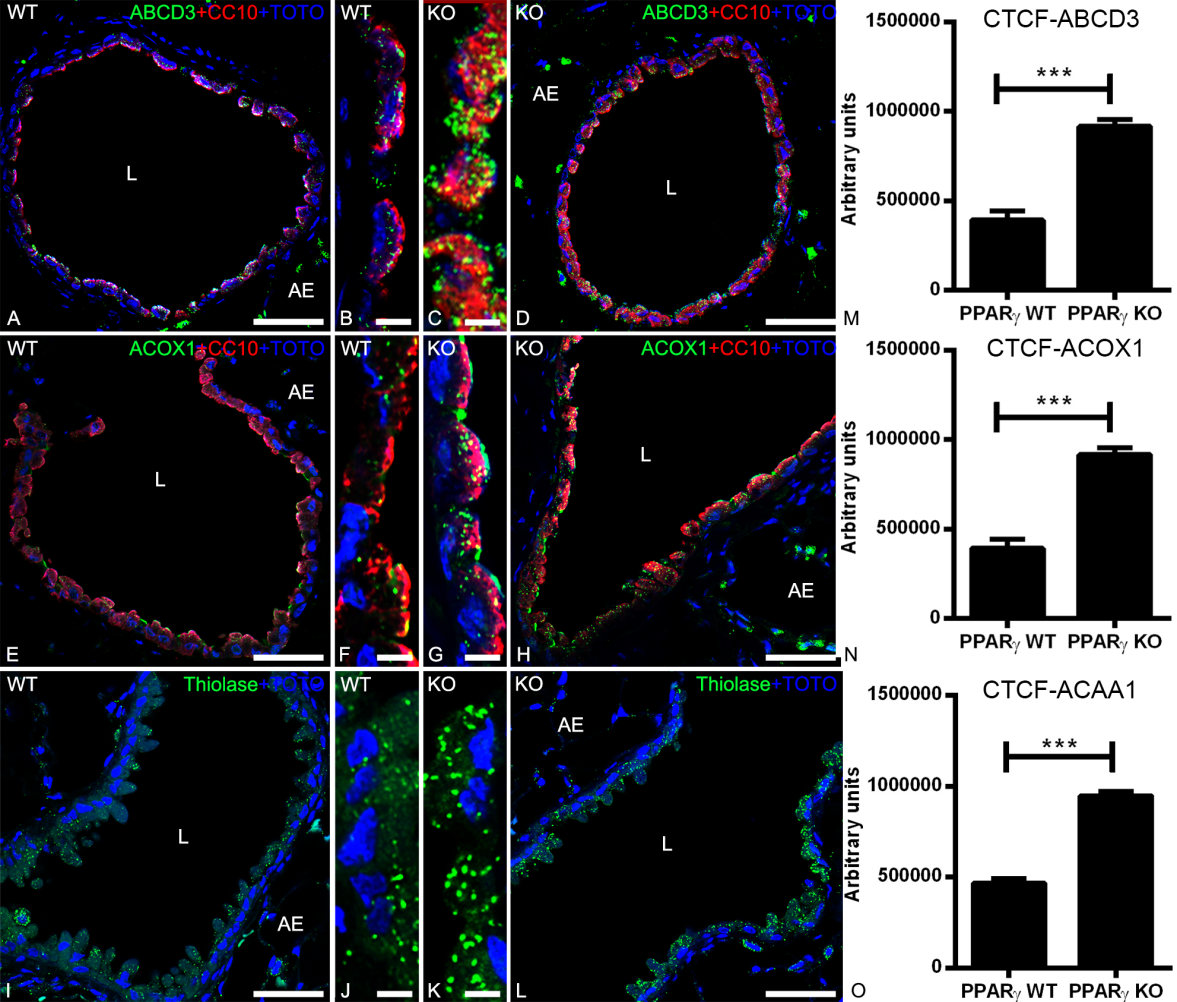
Double-immunofluorescence detection of peroxisomal proteins together with CC10 in the bronchioles of WT and PPARγ-deficient club cells. Staining of mouse lung tissue sections of WT and ccsPPARγKO for peroxisomal lipid transporter ABCD3 (3A-3D) or peroxisomal β-oxidation enzymes ACOX1 (3E-3H) and thiolase (3I-3L). Note that ABCD3 (3C-3D), ACOX1 (3G-3H) and thiolase (3K-3L) protein abundance is increased in PPARγ-deficient club cells. Representative lower (3A, 3D, 3E, 3H, 3I, 3L) and higher magnifications (3B, 3C, 3F, 3G, 3J, 3K) of cross sections of bronchioles in the mouse lung are depicted. Corrected total cell fluorescence (CTCF) quantification of staining for ABCD3 (3M), ACOX1 (3N), and thiolase (3O) in club cells. Values ± SEM represent the mean of CTCF quantified from images obtained from 3 independent experiments using Image J software. **P ≤0.01; ***P ≤0.001; ns, not significant. Counterstaining of nuclei was performed with TOTO-3 iodide. 3L: lumen of the bronchiole; AE: alveolar epithelium. Bars represent: 3A, 3D, 3E, 3H, 3I and 3L: 50 μm; 3B, 3C, 3F, 3G, 3J and 3K: 5 μm.

To investigate whether the PPARγ-deficiency induces alterations in the expression level of proteins involved in the peroxisomal lipid metabolism, we performed IF analyses of WT and ccsPPARγKO lung tissue samples with antibodies against 1) the peroxisomal lipid transporter ABCD3, 2) the first and rate-limiting enzyme of the peroxisomal β-oxidation pathway acyl-CoA-oxidase 1 (ACOX1), bearing a C-terminal PTS1 and imported through PEX5p 3) the last enzyme of this pathway, 3-ketoacyl-CoA-thiolase (AcAA1) bearing a C-terminal PTS2 and imported through PEX7p. Our results showed a significant upregulation of these proteins in the PPARγ-deficient club cells, suggesting an elevation of the peroxisomal lipid-turnover (Fig 3A–3O).

### PPARγ-deficiency in airway epithelial cells induces an increase in peroxisome related gene expression

**Fig 4:**
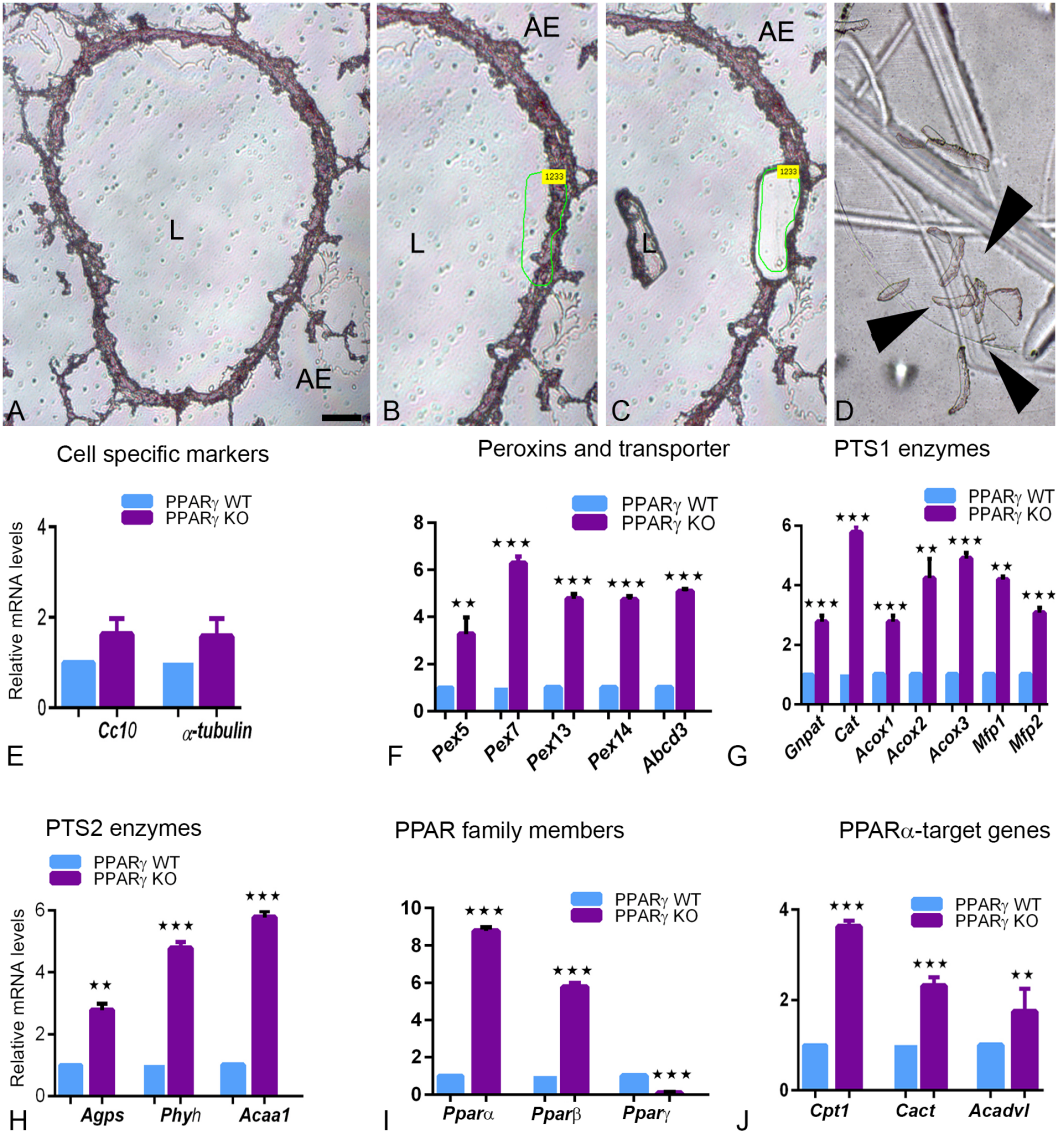
Microdissection of bronchial epithelial cells isolated from WT and ccsPPARγKO lung tissues, and subsequent qRT-PCR analysis for specific mRNAs of peroxisome biogenesis-related and metabolism-related genes, PPARs and mitochondrial PPARα target genes. (4A-4D). Microdissection procedure of frozen lung sections was depicted. WT and ccsPPARγKO sections were stained with hematoxylin (4A), the bronchiolar epithelium was marked (4B) and with the help of laser energy the marked regions cut out and catapulated (4C) into the lid of an Eppendorf tube (4D). qRT-PCR analysis for the amplification of specific mRNAs of club cell secreting protein 10 (CC10) and ciliated cell marker protein α-tubulin (4E), peroxisomal biogenesis proteins (4F), PTS1-containing enzymes (4G), PTS2-containing enzymes (4H) different isoforms of PPAR nuclear receptors (4I) and PPARα target genes (4J). For gene abbreviations and primer sequences see Table 2. The expression of the house keeping gene HPRT was used for normalization. Values ± SEM represent the mean relative fold induction from three independent experiments. *P ≤0.05; **P ≤0.01; ***P ≤0.001. Arrow heads: microdissected regions. L: lumen, AE: alveolar epithelium. Bars represent: 4B-4D: 50 μm.

Because good antibodies against peroxisomal proteins are limited, we decided to examine the mRNA expression levels of multiple genes involved in various different peroxisomal metabolic pathways. By using microdissection pure bronchiolar epithelial cells containing a high population of club cells were isolated from wt and ccsPPARγKO lung tissue as depicted in Fig 4A–4D. Accordingly, the mRNA expression of the CC10 protein, the typical club cell marker, and of α-tubulin, the typical marker for ciliated cells, could be shown by qRT-PCR analysis (Fig 4E). In order to assess the effects of the PPARγ-deficiency on the peroxisomal compartment, we further analyzed the expression of genes encoding proteins involved in PTS1 and PTS2-dependent matrix protein import, different oxidation pathways, plasmalogen synthesis and ROS-metabolism. PPARγ-deficiency induced a strong upregulation of peroxisome-related genes involved in organelle biogenesis and metabolism. In comparison to the tissue derived from wt, mRNAs coding for peroxisomal biogenesis proteins (*Pex5*, *Pex7*, *Pex13* and *Pex14*) and lipid transporter (*Abcd3*) were significantly upregulated in the ccsPPARγKO-derived lung samples (Fig 4F). The results obtained on the protein level by IF staining were fully corroborated by the increased levels of the corresponding mRNAs for*Pex14 and Abcd3 (*Fig 4F). In addition, the mRNAs of the antioxidant enzyme catalase (*Cat*) and of several β-oxidation enzymes (*Acox1*, *Acox2*, *Mfp1*, *Mfp2*, *Acaa1*) were highly upregulated in PPARγ-deficient club cells (Fig 4G). Similarly, the mRNAs of enzymes involved in the peroxisomal ether lipid synthesis (*Gnpat*,*Agps*) and peroxisomal alpha-oxidation of branched fatty acids (*Phyh*) were significantly upregulated (Fig 4H). Hence, PPARγdeficiency significantly upregulates both PTS1 and PTS2 dependent peroxisomal matrix import pathways.

### PPARα and PPARβ mRNAs are significantly upregulated in PPARγ-deficient club cells

Since it is known from the literature that genes encoding for peroxisomal proteins involved in lipid metabolism (ACOX1, MFP1 and ACAA1) are regulated by nuclear receptors of the PPAR family, we checked whether the expression of PPARα and PPARβ were altered due to the PPAR-deficiency. Indeed, we found that PPARγ-deficiency in club cells induced a significant upregulation of PPARα, and to lesser extent also of PPARβ, suggesting a mechanism to compensate for the altered lipid metabolism caused by the PPARγ-deficiency in club cells (Fig 4I). Therefore, we also analysed the expression of PPARα-target genes coding for proteins involved in mitochondrial lipid transport such as those coding for carnitine palmitoyl transferase I (CPTI) and for carnitine acylcarnitine translocase (CACT), controlling mitochondrial β-oxidation flux and for the very long chain acyl-CoA dehydrogenase (ACADVL). All mitochondrial PPARα target genes were significantly upregulated in ccsPPARγKO lungs, however, to a lesser extent than the peroxisomal ones (Fig 4J).

### PPARγ knockdown induces upregulation of PPARα and proteins involved in peroxisomal biogenesis and lipid metabolism in C22 cells

**Fig 5:**
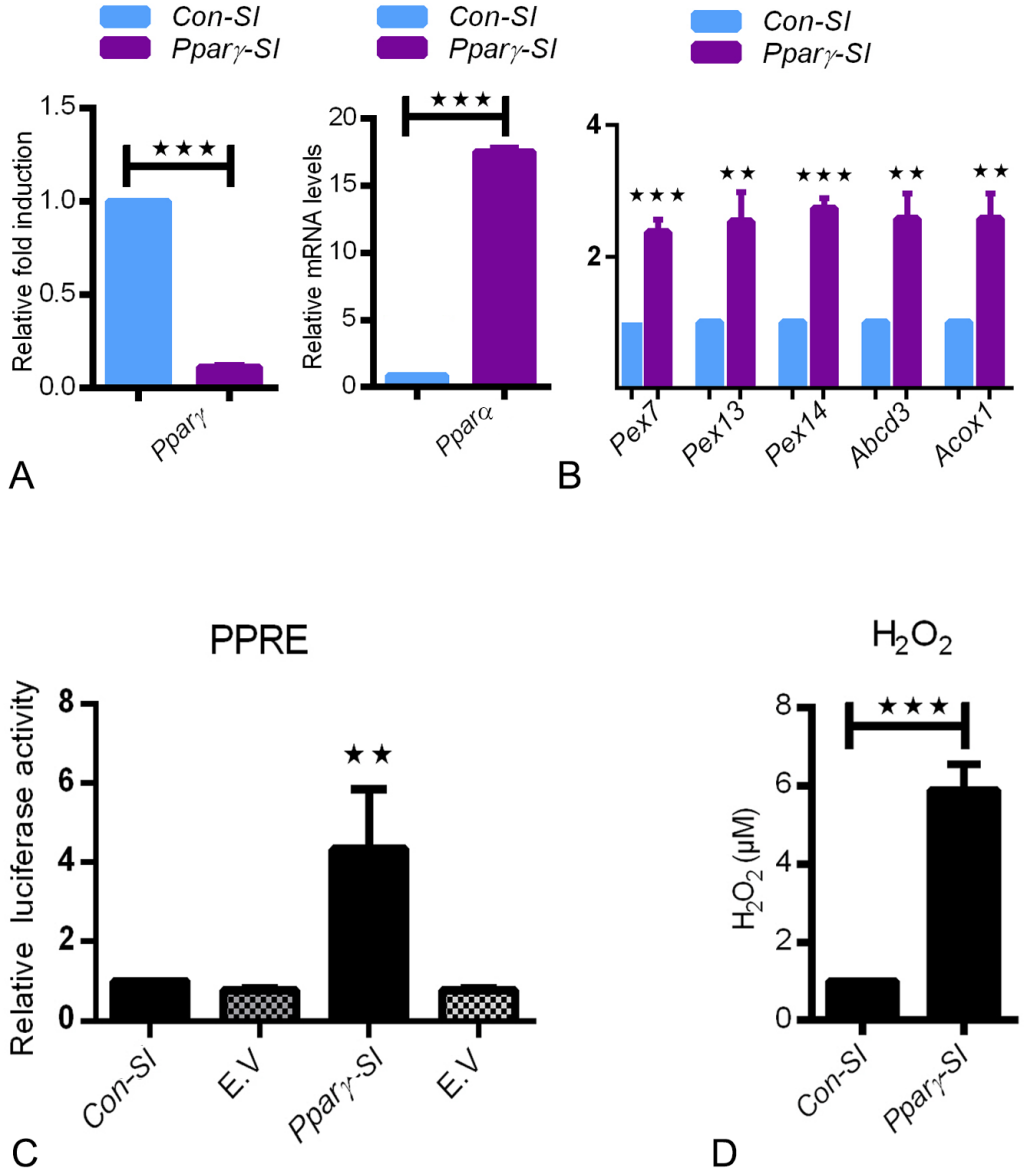
C22 cells were transfected with *control*-siRNA (Con-SI) or *pparγ*-siRNA (Pparγ-SI) for 48h. The knockdown was verified by qRT-PCR for Pparγ(A). Further, qRT-PCR analysis was performed for Pparα (5A), mRNAs for peroxisomal biogenesis proteins such as *Pex7p*, *Pex13p*, *Pex14p* and lipid transporter *Abcd3* as well as peroxisomal β-oxidation enzyme *Acox1* (5B). The expression of the house keeping gene HPRT was used for normalization. Values ± SEM represent the mean relative fold induction from three independent experiments. *P ≤0.05; **P ≤0.01; ***P ≤0.001. Dual luciferase reporter activity of PPRE was measured in C22 cells treated either with *control*-siRNA (Con-SI) or *pparγ*-siRNA (Pparγ-SI). The activity of luciferase was measured in cell lysates and normalized to the activity of renilla. (E.V-empty vector) (5C). Data represent ± SD of three independent experiments, *P* value, unpaired Student t-test. H_2_O_2_ assay was performed as per manufacture instructions (5D).

To simulate the in vivo findings and delineate the PPARγdeficiency in club cells, we employed an *in vitro* approach by using siRNA-mediated *PPAR*γ knockdown in cultured C22 cells. siRNA-mediated PPARγ-deficiency in C22 cells also showed a significant upregulation of *PPAR*α, increased the expression of peroxisomal biogenesis proteins (*Pex7*, *Pex13*, *Pex14*), lipid transporter (*Abcd3*), and the rate-limiting peroxisomal β-oxidation enzyme (*Acox1*) fully corroborating the *in vivo* observations of PPARγ-deficient microdissected bronchial epithelial cells (Figs 5A, 5B, 4F and 4G). Further, the knockdown of PPARγ in club cells also induced a significant increase in the PPRE activity and a significant increase in the mRNA expression of PPARα (Fig 5A–5C). Moreover, active release of H_2_O_2_ was detected in PPAR-deficient C22 cells in comparison to the control cells suggesting that PPARγ deficiency induces oxidative stress.

Similarly, PPARγ inhibition with the specific antagonist GW9662 also increased the expression of PPARα and the activity of the PPRE reporter plasmid (Figure A and B in S1 Fig). In parallel experiments, inhibition of PPARγ with GW9662 also showed increased H_2_O_2_ release, in agreement to our previous observations (Figure C in S1 Fig and Fig 5D). Taken together, luciferase-reporter assays and mRNA expression studies in C22 cells revealed that the PPARγ selective antagonist GW9662 or siRNA-mediated knockdown of PPARγ activated the PPRE-mediated luciferase expression and induced the upregulation of PPARα and peroxisomal gene transcription.

### PPARγ-deficiency in club cells leads to accumulation of neutral lipids in lung homogenates

**Fig 6:**
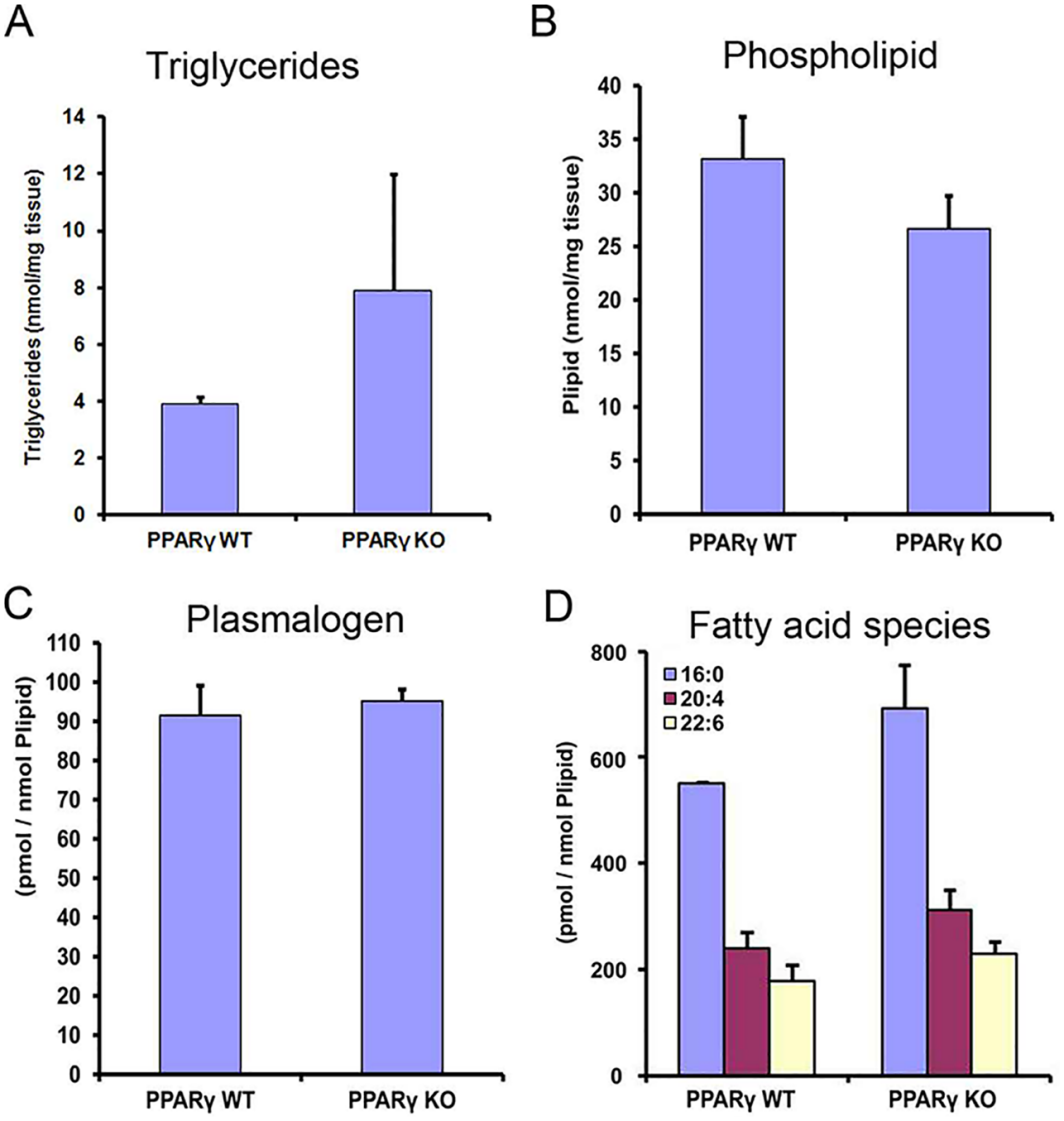
Lipid profiling from WT and ccsPPARγKO lung tissues analyzed for triglycerides (6A) phospholipids (6B), plasmalogens (6C) and fatty acid species (6D). Palmitic acid (C16:0), arachidonic acid (C20:4) and docosahexaenonic acid (DHA; C22:6).

PPARα activation is often associated with changes in lipid metabolism [5]. To determine whether changes in the composition or quantity of intracellular lipids occurred in ccsPPARγKO lungs, we have quantified triglycerides, total phospholipids, plasmalogens and overall fatty acid composition from total lipid extracts. Interestingly, the total triglyceride nmol per milligram lung tissue was 2 fold higher in the homogenate derived from the ccsPPARγKO lung tissue in comparison to wt tissue (Fig 6A). In contrast, the total phospholipid nmol per milligram of lung tissue did not display major differences between the wt and ccsPPARγKO homogenates although a trend for the PPARγ-deficient lung-homogenates to contain less phospholipid was evident (Fig 6B). Moreover, no difference in the plasmalogens content could be detected between lung homogenates derived from wt and those derived from ccsPPARγkO lungs (Fig 6C). Furthermore, the total percentage of different fatty acid species [such as palmitic acid (C16:0), arachidonic acid (C20:4) and docosahexaenoic acid (DHA; C22:6)] were increased in the ccsPPARγkO tissue in comparison to wt tissue (Fig 6D).

## Discussion

The peroxisome proliferator activated receptor γ (PPARγ) is an emerging anti-inflammatory, anti-oxidative protein and plays an important role in a variety of lung diseases such as asthma and COPD [3]. Although PPARγis widely expressed in all pulmonary cell types its function is cell-type specific [3]. To analyse the specific function of PPARγin club cells Mariani’s group generated a ccsPPARγKO mouse [6]. CcsPPARγKO mice showed insufficient postnatal lung maturation and epithelial cell differentiation and exhibited an increase in the air space size in the adult lung. Mariani’s group also performed a genome-wide microarray expression analysis of isolated airway-epithelial cells. Interestingly, the *Pex7* gene encoding the receptor for PTS2-targeted peroxisomal matrix protein was found amongst the 10 most upregulated genes [6]. This finding was corroborated by the qRT-PCR analysis of microdissected bronchiolar airway epithelial samples and siRNA-mediated PPARγ knockdown in C22 cells presented in this study. Similar results were also observed in the primary Osteoblasts that were treated with PPARγ antagonist, GW9662 increased PEX13p protein abundance [18].

Moreover, we found that the PPARγ-deficiency in club cells induced the biogenesis of peroxisomes, and the abundance of PTS1 and PTS2 containing proteins in the peroxisomal matrix. It further upregulated the expression of mRNAs and the abundance of proteins involved in the peroxisomal β-oxidation. PTS1 bearing proteins are recognized and bound by the cytoplasmic receptor protein PEX5p, whereas PTS2 bearing proteins are recognized by the receptor PEX7p. Both proteins were induced by the PPARγ-deficiency [19, 20]. After binding their cargo protein in the cytoplasm PEX5p or PEX7p interact with the peroxisomal docking and translocation machinery [19], a membrane protein complex comprising PEX13p, PEX14p and PEX12p to translocate the cargo to the peroxisome matrix [5, 19, 20]. PEX14p is the best marker protein of mouse and human peroxisomes in all different pulmonary cell types [4, 21]. In this study, Pex14p labeled large peroxisomes in club cells and small peroxisomes in ciliated cells of the bronchiolar epithelium confirming our previous results [4]. This staining also revealed the proliferation of peroxisomes in ccsPPARγKO lungs. Also catalase was strongly upregulated at both the mRNA and the protein level in PPARγ-deficient club cells to degrade released H_2_O_2_. Indeed, *in vitro* experiments showed elevated levels of H_2_O_2_ suggesting that catalase played protective role in reducing the oxidative stress by its upregulation. However, even strong upregulation of catalase in degrading H_2_O_2_ might lead to mistargeting suggesting an adaptation of peroxisomal compartment to increased oxidative stress. The strong upregulation of the expression of the gene coding for catalase could be a result of the observed increase in the expression of PPARα. Indeed, previous studies reported that PPARα is able to bind to the PPRE element in the catalase promoter in absence of PPARγ [22, 23].

Further the expression and the subcellular localisation of catalase was shown to be regulated by PPARγ, NfkB and oxidative stress [24]. It is interesting that in our study, catalase was partially mistargeted to the cytoplasm in ccsPPARγKO mice, an effect, which is known to occur under oxidative stress conditions [25] and is probably a result of the weakend interaction to its receptor PEX5p [26]. PEX5p is a redox-sensitive protein, wherefore oxidative stress induced by PPARγ-deficiency might induce catalase mistargeting [27].

In addition to the expression of catalase, also the expression of other PPARα target genes was increased, including the one of peroxisomal lipid transporter and enzymes of the peroxisomal β-oxidation pathway. Similarly, in mitochondria, CPT1, involved in fatty acid transport and VLCFA mitochondrial β-oxidation displayed increased expression levels. Given that PPARα mediated induction of peroxisomes in hepatocytes in rodents, PPARα activation might also be responsible for the peroxisome proliferation in lung [28].

The transcriptional regulation of the genes coding for proteins of the peroxisomal compartment by the activation of PPARα by endogenous fatty acid ligand was previously reported, however, how this occurs in airway epithelial cells when PPARγ is deleted remains unclear [2, 29, 30]. Interestingly, we observed an increase of fatty acids [palmitic acid (C16:0), arachidonic acid (C20:4) and docosahexaenonic acid (C22:6)]. The increase in C16:0 is likely related to accumulation of triglycerides, whereas changes in PUFA suggest alterations in membrane phospholipids composition and fluidity. Existing literature indicates that the release of these fatty acids from phospholipids might generate physiological ligands for PPARα. Long chain fatty acids, saturated and poly-unsaturated fatty acids have been shown to bind and activate PPARα even at micromolar concentrations [2, 29–33]. These ligands bind to PPARα, stimulate its heterodimerization with RXR and its nuclear translocation and induce proliferation of peroxisomes [32–34]. PPARα induction leads to the transcriptional activation of genes coding for peroxisomal β-oxidation enzymes [35] and this pathway in turn is involved in the control of the homeostasis of PPAR ligands [4, 5]. Our results showed that a PPRE-mediated transcriptional activation of PPARα increases the number of peroxisomes and metabolic activity in C22 cells. Further, PPARα also induces genes controlling the fatty acid import into the mitochondria (CPT1 and 2), as well as the major enzymes within the β-oxidation pathway including various acyl-CoA dehydrogenases (ACADs), which were also induced in the PPARγ deficient lung club cells suggesting the involvement of PPARα in the transcriptional regulation of mitochondrial enzymes. In this respect, it is of interest that peroxisomes and mitochondria cooperate in the degradation of fatty acids and peroxisomes will compensate mitochondrial fatty acid oxidation of palmitate in case the mitochondrial CPT1 system is saturated and overloaded [5, 36].

We assume that, increased palmitic acid levels, a fatty acid, whose affinity for PPARα-binding is high (IC values ranging from 1.1–5.4 μM) promotes the PPARα gene transcription [37]. In this respect, it is of interest that PPARα can stimulate the transcription of its own gene by an autocrine loop. Using scintillation proximity binding assays, Xu and colleagues revealed that saturated and unsaturated fatty acids containing 16 carbons bind PPARα, only very weakly to PPARβ and failed to bind to PPARγ [37]. Using a computational approach, six different scoring programs were used to analyze the binding affinity and docking of DHA to PPARs showing that PPARα was able to bind this lipid derivate. Moreover, in another study based on the binding efficiency and torsion angles as well as docking sites of DHA binding to PPARs showed that DHA docked with higher affinity to PPARα than PPARγ. Based on the literature and our results we suggest that, PPARγ-deficiency is compensated by the upregulation of PPARα and its target genes.

**Fig 7:**
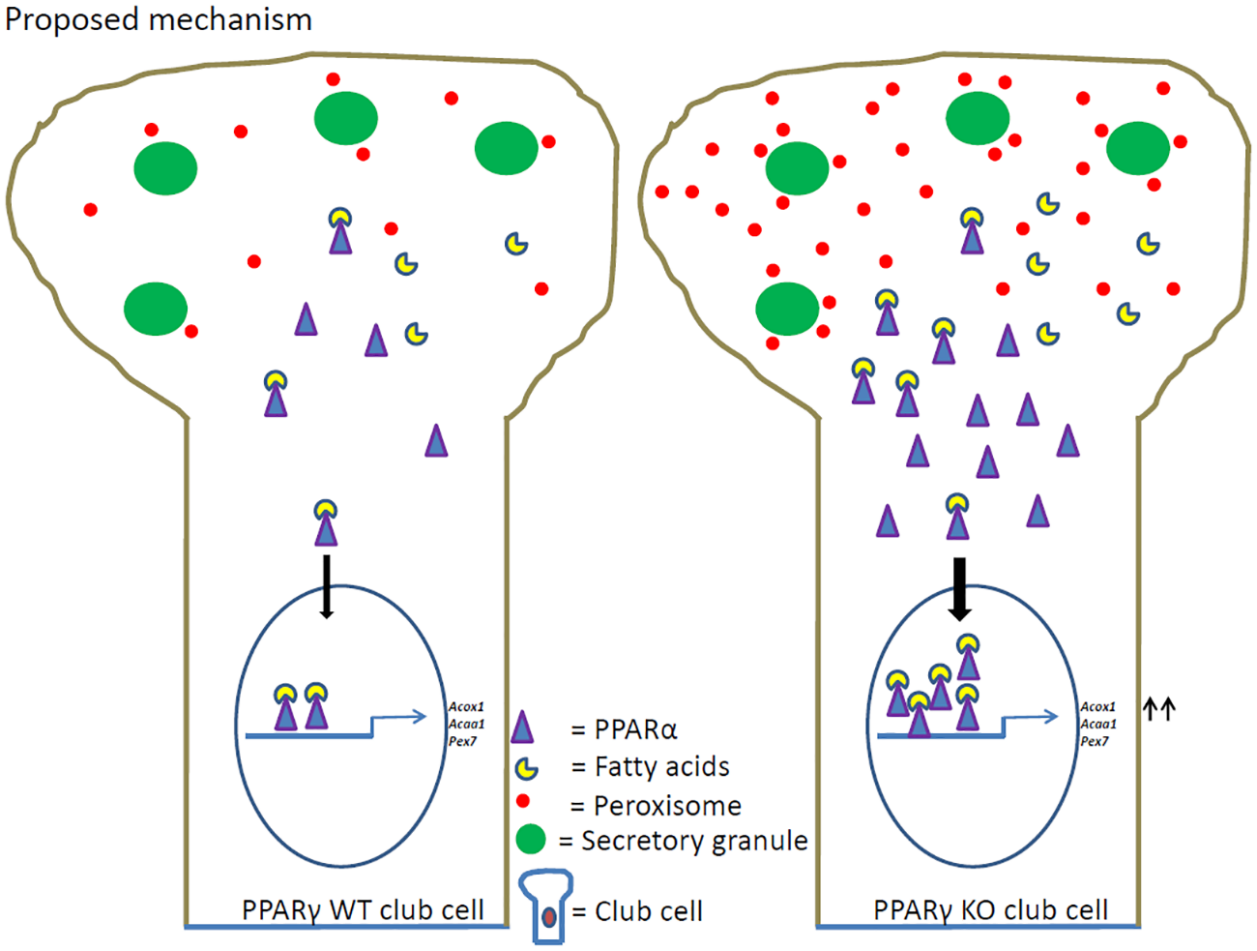
Proposed mechanism of peroxisome induction in club cells with PPARγ-deficiency. In WT club cell, endogenous fatty acids are ligands for nuclear receptor PPARα and its basal transcription of peroxisomal genes. However, PPARγ-deficiency in club cells leads to increased amounts of palmitic acid (C16:0), arachidonic acid (C20:4) and docosahexaenoic acid (DHA; C22:6) in phospholipids. The release of free fatty acids might generate physiological ligands for the nuclear receptor PPARα. PPARα acts as a transcription factor, forming heterodimers with the retinoid X receptor which binds to specific response elements (PPREs) in the promoter regions of the peroxisome-related genes (*Pex7*, *Acox1* and *Acaa1*). PPARα induction leads to orchestrated upregulation of gene expression for proteins regulating peroxisomal biogenesis and metabolism leading to peroxisome proliferation and enzyme induction to protect the airway epithelium against oxidative and lipotoxic stress.

Taken together, our results suggest that PPARγ-deficiency in club cells of the airway epithelium induces peroxisome proliferation leading to alterations of peroxisomal compartment and increased fatty acids in the phospholipid fraction. These increased fatty acids in phospholipids might generate physiological ligands for PPARα. PPARα regulates the numerical abundance and metabolic function of peroxisomes via a PPRE-dependent mechanism. PPARα is known to bind to the promoter regions of the peroxisomal genes *Pex7*, *Acox1* and thiolase thereby regulating transcription of peroxisomal β-oxidation genes as well as in different pathways to protect the airway epithelium against ROS and lipid toxicity, preventing major damage of the lung structure (Fig 7).

## Supporting information

S1 Fig GW9662 treatment in C22 cells induces PPRE activity and PPARα upregulation. C22 cells were treated with PPARγ antagonist (GW9662) for 24 h with a concentration of 5 μM. Total RNA was isolated from these cultures and subjected to qRT-PCR analysis for PPARα (A). The expression of the house keeping gene HPRT was used for normalization. Values ± SEM represent the mean relative fold induction from three independent experiments. **P ≤0.01; ***P ≤0.001. Dual luciferase reporter activity of PPRE was measured in C22 cells treated either with control (Con) or GW9662 (B). The activity of luciferase was measured in cell lysates and normalized to the activity of renilla. (E.V-empty vector). Data represent ± SD of three independent experiments, *P* value, unpaired Student t-test. The culture supernatants were collected subjected to H_2_O_2_ assay as per manufacture instructions (C).
(DOCX)

S2 Fig Immunofluorescence analysis revealed that CC10-Cre expression alone did not induce peroxisome proliferation and peroxisomal alterations. We assumed that the *Cre*-expression itself in lung club cells might alter the peroxisome compartment as Cre-expression in Sertoli cells of the testis already induced severe alterations in peroxisome biogenesis and proteome. Therefore, immunofluorescence was performed with PEX14p (A-C) and ACAA1 (D-F) in WT, CC10-*Cre*-, WT transgenic animals in comparison to heterozygous (PPARγ-LoxP animals). Interestingly, in Cre-expressing-transgenic animals the peroxisomal protein composition was not altered compared to wt lungs suggesting that the Cre-mediated effects are cell-type-specific. Corrected total cell fluorescence (CTCF) quantification of staining for PEX14p (G) and thiolase (H) in club cells of transgenic mice in comparison to WT mice. Values ± SEM represent the mean of CTCF quantified from images obtained from 3 independent experiments using Image J software. **P ≤0.01; ***P ≤0.001; ns, not significant. Representative higher magnifications of cross sections of bronchioles in the mouse lung are depicted. L: lumen of the bronchiole; AE: alveolar epithelium; * represent the cross-staining of erythrocytes. Bars represent A-F: 20 μm.
(DOCX)

## Abbreviations

AECII: alveolar epithelial cell type II
CC10: club cell secretory protein 10
COPD: chronic obstructive pulmonary disease
RXR: retinoid X receptor
