# Supplementary figures and images for: PPARα-mediated peroxisome induction compensates PPARγ-deficiency in bronchiolar club cells
Source: PLoS One. 2018 Sep 13;13(9):e0203466. doi: 10.1371/journal.pone.0203466 (PMC6136741; doi:10.1371/journal.pone.0203466)

**
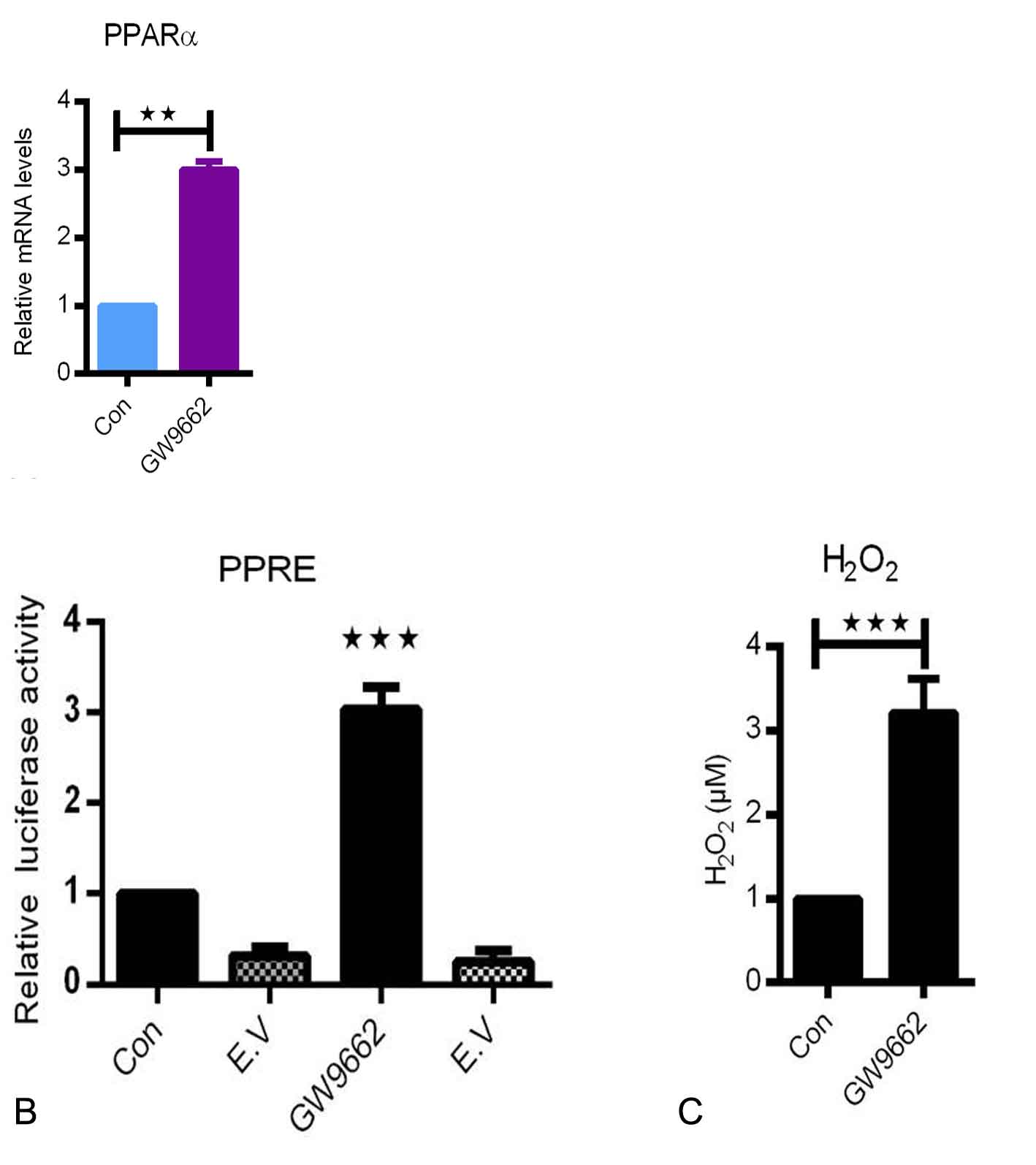
**

**A**

Supplement: S1 Fig — Total RNA was isolated from these cultures and subjected to qRT-PCR analysis for PPARα (A). The expression of the house keeping gene HPRT was used for normalization. Values ± SEM represent the mean relative fold induction from three independent experiments. **P ≤0.01; ***P ≤0.001. Dual luciferase reporter activity of PPRE was measured in C22 cells treated either with control (Con) or GW9662 (B). The activity of luciferase was measured in cell lysates and normalized to the activity of renilla. (E.V-empty vector). Data represent ± SD of three independent experiments, P value, unpaired Student t-test. The culture supernatants were collected subjected to H2O2 assay as per manufacture instructions (C). (DOCX) [file pone.0203466.s001.docx]

**
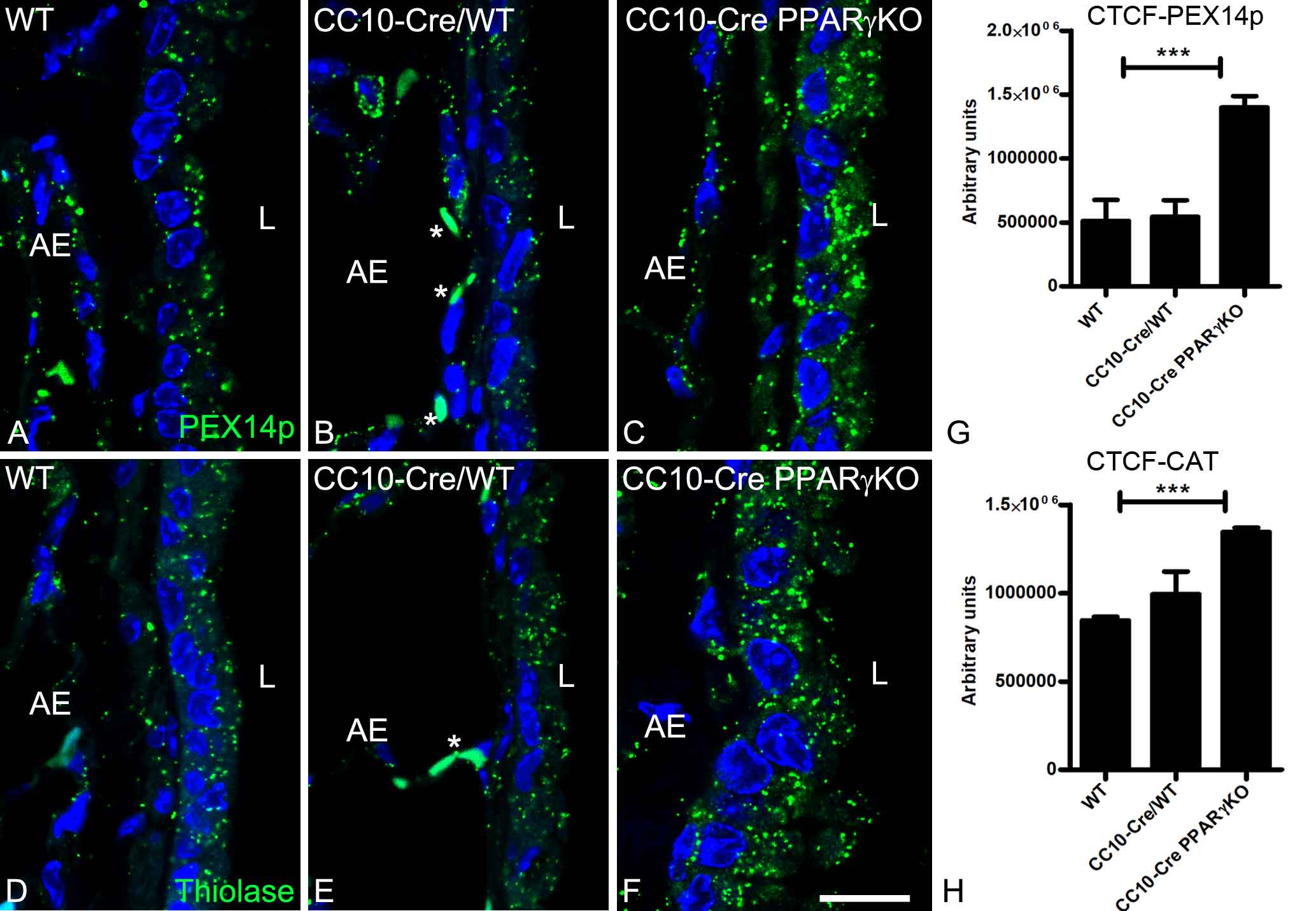
**

Supplement: S2 Fig — We assumed that the Cre-expression itself in lung club cells might alter the peroxisome compartment as Cre-expression in Sertoli cells of the testis already induced severe alterations in peroxisome biogenesis and proteome. Therefore, immunofluorescence was performed with PEX14p (A-C) and ACAA1 (D-F) in WT, CC10-Cre-, WT transgenic animals in comparison to heterozygous (PPARγ-LoxP animals). Interestingly, in Cre-expressing-transgenic animals the peroxisomal protein composition was not altered compared to wt lungs suggesting that the Cre-mediated effects are cell-type-specific. Corrected total cell fluorescence (CTCF) quantification of staining for PEX14p (G) and thiolase (H) in club cells of transgenic mice in comparison to WT mice. Values ± SEM represent the mean of CTCF quantified from images obtained from 3 independent experiments using Image J software. **P ≤0.01; ***P ≤0.001; ns, not significant. Representative higher magnifications of cross sections of bronchioles in the mouse lung are depicted. L: lumen of the bronchiole; AE: alveolar epithelium; * represent the cross-staining of erythrocytes. Bars represent A-F: 20 μm. (DOCX) [file pone.0203466.s002.docx]
